# A Highly Transparent Thermoplastic Synthesized from Ethylene that Melts Above 200 °C

**DOI:** 10.1002/anie.202505834

**Published:** 2025-08-17

**Authors:** Fabian Lukas, Andre Dickert, Winfried P. Kretschmer, Rhett Kempe

**Affiliations:** ^1^ Lehrstuhl für Anorganische Chemie II–Katalysatordesign, Sustainable Chemistry Centre University of Bayreuth Bayreuth 95440 Germany

**Keywords:** High melting point, High transmittance, Polyethylene, Thermoplastic, Very low density

## Abstract

Polyolefin‐based thermoplastics are the most widely used polymers and produced in megaton scale. Their applicability depends strongly on the melting point, and a high‐melting ethylene‐based plastic would be highly desirable. Herein, we report the synthesis of *isotactic* poly(4‐ethylhex‐1‐ene) (P4EH), a highly transparent and ethylene‐based thermoplastic that melts above 220 °C and has an extremely low density. Molecular catalysts were used to synthesize *atactic*, *syndiotactic*, and *isotactic* P4EH with narrow molecular weight distributions. Ziegler–Natta catalysts were used to synthesize *isotactic* and processable P4EH. Processable *i*‐P4EH was used to produce discs, and a transmittance of 92%, a haze of 8% and a clarity of 92% were measured. In addition, a density of 0.86 g cm^−3^ was observed.

## Graphical abstract

We introduce a highly transparent ethylene‐based thermoplastic (transmittance of 92%, haze of 8% and clarity of 92%) with a very low density (0.86g cm^−3^) and an high melting point (up to 228 °C).

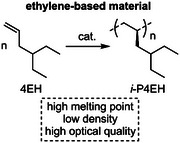

## Introduction

**Figure 1:**
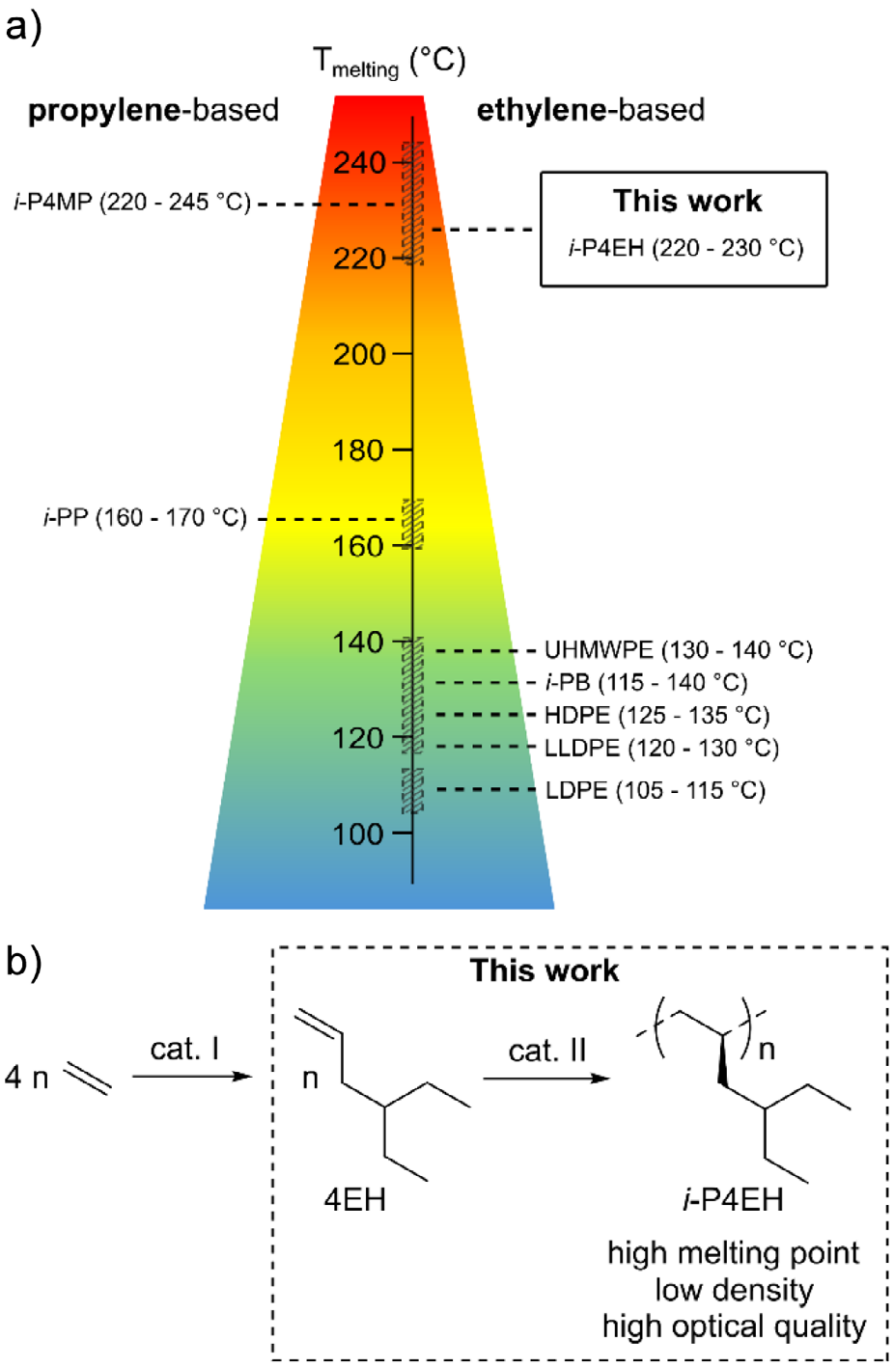
State of the art and work reported here. a) Ethylene‐ and propylene‐based thermoplastics and their melting point ranges. b) Synthesis of *i*‐P4EH from ethylene reported here.

Ethylene‐based plastics, such as high‐, low‐, and linear low‐density polyethylene (HDPE, LDPE, and LLDPE, respectively) are produced on a megaton scale annually and account for about one third of the world's plastic production volume and market.^[^ ^1^, ^2^ ^]^ The inexpensive and abundantly available feedstock ethylene, which can be derived from many resources,^[^ ^3^, ^4^, ^5^ ^]^ including biobased resources (via fermentation), is the basis of the impressive applicability of these plastics. The HDPE, LDPE, and LLDPE are thermoplastics, and the melting point is a key property. An ethylene‐based plastic with a high‐melting point would be highly desirable. Optical properties are also important, and high transparency and a low haze of polyethylene materials are of general interest. We recently^[^ ^6^ ^]^ reported the elongation and branching of α‐olefins by two ethylene molecules. After investigating different catalysts,^[^ ^7^, ^8^ ^]^ we discovered the tetramerization of ethylene towards 4‐ethylhex‐1‐ene (4EH) based on the elongation‐branching reaction without polymer byproduct formation even after 3 h.^[^ ^8^ ^]^ The 4EH had been synthesized previously via a seven‐step catalytic organic synthesis involving hydroformylation.^[^ ^9^ ^]^ Herein, we report the synthesis of *isotactic* poly(4‐ethylhex‐1‐ene) (*i‐*P4EH), a highly transparent ethylene‐based thermoplastic with a very high melting point (Figure 1a).^[^ ^10^ ^]^ Molecular catalysts were used to synthesize *atactic*, *syndiotactic*, and *isotactic* P4EH with narrow molecular weight distributions and Ziegler–Natta catalysts were used to synthesize *isotactic* and processable P4EH with a broader polydispersity (Figure 1b). The melting points of the *isotactic* P4EH samples lay between 223 and 229 °C. Processable *i*‐P4EH was used to produce discs and a transmittance of 92%, a haze of 8% and a clarity of 92% were measured. In addition, a density of 0.86 g cm^−3^ was observed for *i*‐P4EH. For the melting points of propylene‐based plastics, such as *isotactic* polypropylene (*i*‐PP)^[^ ^10^ ^]^ and *isotactic* poly(4‐metylpent‐1‐ene) (*i*‐P4MP),^[^ ^11^ ^]^ see Figure 1a.

## Results and Discussion

**Figure 2:**
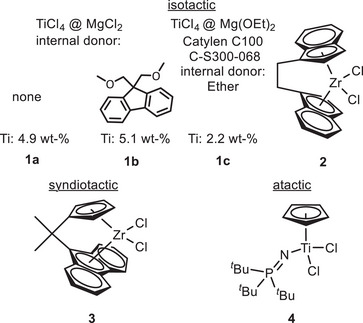
Selected precatalysts for the isotactic (**1a**, **1b**, **1c**, and **2**), syndiotactic (**3**), and atactic (**4**) polymerization of 4EH.

The polymerization of 4EH was studied using six different heterogeneous or homogeneous (pre)catalysts (Figure 2). Isotactic polymers were obtained with either a 2^nd^ generation Ziegler–Natta (ZN) system **1a**, a 5^th^ generation Ziegler–Natta^[^ ^12^, ^13^ ^]^ (ZN) system **1b** and **1c** or a Brintzinger‐type C_2_‐symmetric *ansa* zirconocene^[^ ^14^ ^]^ **2**. *Ansa* zirconocene^[^ ^15^ ^]^ **3** with C_S_ symmetry and half titanocene^[^ ^16^ ^]^ **4** with an additional phosphinimide ancillary ligand were used to prepare syndiotactic and atactic samples, respectively. The precatalysts were activated with different cocatalysts and tested at different temperatures (Table 1).

**Table 1:** Polymerization of 4EH using precatalysts **1**–**4** with various activators and at different reaction temperatures.^a)^ ^.^

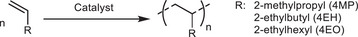									
Entry	Monomer	Catalyst	*T* (°C)	Conversion (mol%)	Yield [mg]	*M*_w_^SEC^ (kg mol^−1^)	Ɖ	*T*_m_^DSC^ ^b)^ (°C)	TOF (min^−1^)
1	4EH	1a/TIBA	50	15	101	19.6	4.9	/	2.0
2	4EH	1b/TIBA	50	22	148	248.8	11.0	226	2.9
3	4EH	1c/TIBA	50	86	579	662.7	21.2	206	11.5
4	4EH	2/TIBA/borate	0	28	189	13.3	2.0	222	0.8
5	4EH	2/TIBA/borate	30	45	303	12.4	2.0	216	1.3
6	4EH	2/AlEt_3_/borate	0	26	175	7.0	1.8	224	0.7
7	4EH	2/MAO	0	30	201	4.5	1.6	223	0.9
8	4EH	3/TIBA/borate	0	10	67	10.7	1.8	111	0.3
9	4EH	3/TIBA/borate	30	33	222	8.4	1.9	/	0.9
10	4EH	4/TIBA/borate	60	18	121	1.6	1.9	/	0.1
11	4MP	2/TIBA/borate	0	54	364	13.8	1.9	224	1.5
12	4EO	2/TIBA/borate	0	15	101	13.2	1.9	92	0.4

^a)^ Reaction conditions: Precatalyst **1**: *n*(Ti)  =  15 µmol, activator: 15 equiv. triisobutylaluminum (TIBA), *t*  = 0.5 h, n(monomer)  =  6 mmol, solvent: toluene, Σ *V*  = 5 mL. Precatalysts **2** and **3**: *n*(Zr)  =  5 µmol, activator: 1.1 equiv. ammonium borate ([R_2_N(CH_3_)H]^+^[B(C_6_F_5_)_4_]^−^, R  =  C_16_H_33_ to C_18_H_37_) or 500 equiv. methylalumoxane (MAO), scavenger and alkylating agent: 10 equiv. TIBA, *t*  =  7 h, n(monomer)  =  6 mmol, solvent: toluene, Σ *V*  =  5 mL. Precatalyst **4**: analogous to **2** and **3** with *n*(Ti)  =  20 µmol.

^b)^ Highest melting point. DSC melting and crystallization endotherms can be found in Supporting Information Figures S8–S12 and S24–S27.

### Productivity

The ZN catalytic system **1c** displayed the highest productivity at 50 °C with 86 mol% conversion (Table 1, Entry 3). For **1a** and **1b** (Table 1 Entry 1–2) much lower conversions of 15 and 22 mol% were observed, respectively. All homogeneous catalytic systems studied displayed rather low productivity and needed long reaction times, such as 7 h, to obtain a significant conversion under the experimental conditions applied. 10 equivalents of the alkylating agent TIBA were sufficient to ensure adequate activation. Lowering the amount (5 equivalents, Table S3, Entry 14) resulted in a decreased productivity, whereas an increase (50 equivalents, Table S3, Entry 16) did not yield a more productive system. The usage of 500 equivalents MAO as an activator led to a similar conversion. The productivities were temperature‐dependent with a medium temperature (30–50 °C) being more favorable for all systems (Tables S2–S4,S6). Furthermore, the monomer conversion decreased with the increasing molecular weight of the monomer used from 54 (4MP) to 15% (4‐ethyloct‐1‐ene, 4EO) using **2**/TIBA/borate under identical reaction conditions (Table 1, Entries: 4, 11–12).

### Product Distribution

Lower polymerization temperatures generally resulted in the formation of higher molecular weight polymers for all catalyst systems. The ZN catalyst systems with triisobutylaluminum (TIBA) as the activator gave the highest molecular weight *i*‐P4EH at 50 °C with *M*_w_  =  662.7 kg mol^−1^ for precatalyst **1c**, which decreased to 19.6 and 248.8 kg mol^−1^ for **1a** and **1b**, respectively (Table 1, Entries 1–3). The product distribution *Ɖ* was broad with 4.9 – 21.2, which is expected when using a heterogeneous catalyst with several different catalytic sites. Changing the activator from TIBA to AlEt_3_ resulted in shorter polymer chains, which could be explained by a higher chain transfer rate to AlEt_3_ compared to TIBA or the generation of a different set of active polymerization sites (Table S2, Entry 2). No olefinic resonances were detected in the ^1^H NMR spectrum, indicating the absence of β‐H elimination as the termination reaction (Figure S18). The use of **2** as a homogeneous *single‐site* catalyst (after activation) led to narrowly distributed, low molecular weight polymers with the highest *M*_w_ of 13.3 kg mol^−1^ at 0 °C (Table 1, Entries 4–7). The ^1^H‐NMR analysis revealed internal olefins (Figure S29) resulting from a 2,1‐misinsertion of one monomer unit followed by a termination reaction. This 2,1‐insertion may have contributed to the rather low molecular weight and is documented for metallocene catalysts.^[^ ^17^ ^]^ Activation with AlEt_3_/borate or MAO, (Table 1, Entries 6 and 7) instead of TIBA/borate produced even lower molecular weight chains. This behavior suggests that the chain transfer to Al may also be a relevant chain termination reaction. Catalyst systems consisting of **3**/TIBA/borate behaved similarly to **2** and polymers with *M*_w_ between 8.4 and 10.7 kg mol^−1^ were obtained (Table 1, Entries 8 and 9). **4**/TIBA/borate produced oligomeric chains (*M*_w_: 1.6 kg mol^−1^, Table 1, Entry 10) at 60 °C, which were used for analytical purposes (see NMR section). Internal olefins could be detected for systems **3** and **4** (analogous to **2**). Again, a 2,1 misinsertion of one monomer unit and a subsequent termination reaction can lead to these internal olefins. *Isotactic* P4MP^[^ ^18^ ^]^ (poly(4‐methylpent‐1‐ene) and P4EO (poly(4‐ethyloct‐1‐ene) were synthesized with **2**/TIBA/borate for comparison. The *M*_w_ of these polymers did not differ significantly from *i*‐P4EH, but the corresponding average monomer units per chain decreased with the increasing molecular weight of the monomer from 167 (4MP) to 97 (4EO). All molecular distributions can be found in the Supporting Information (Tables S2–S4; Figures S1–S7, S20–S23; S31,S32).

**Figure 3:**
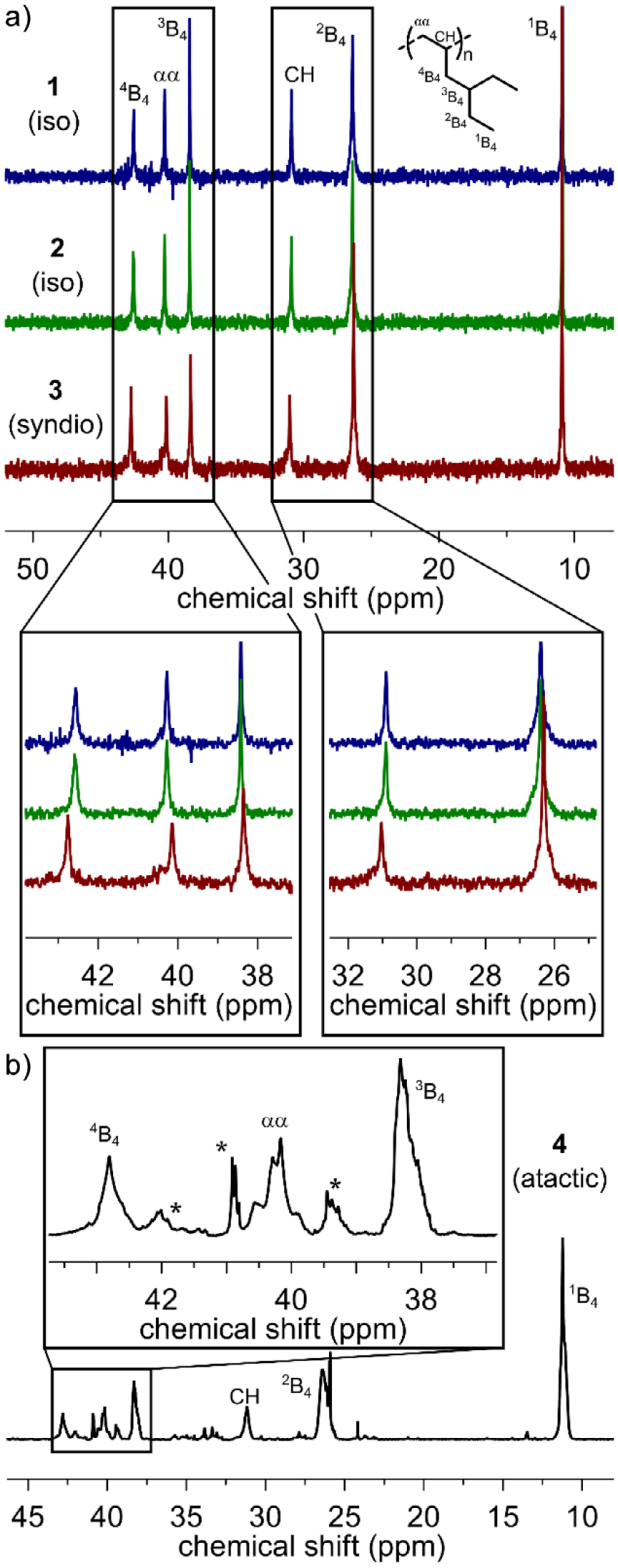
^13^C‐NMR spectra of P4EH. a) Comparison of stereoregular samples synthesized with **1b, 2, 3** (75 MHz, 120 °C, C_2_D_2_Cl_4_). b) Atactic sample synthesized with **4** and measured with a 1 GHz NMR spectrometer (^13^C: 250 MHz, 23 °C, C_6_D_6_). The asterisks mark end group and regio‐defect signals.

**^13^C NMR**: Using ^13^C‐NMR spectroscopy, it was possible to elucidate the microstructure of the polymer.^[^ ^19^, ^20^, ^21^, ^22^ ^]^ A comparison of ^13^C NMR spectra (75 MHz, 120 °C, C_2_D_2_Cl_4_) for P4EH synthesized with **1b** at 50 °C, **2** and **3** at 0 °C and **4** at 60 °C (Table 1, Entries 2, 4, 8 and 10) is presented in Figure 3a. From the polymers yielded with ZN catalysts only one produced with **1b** was chosen, due to their small polydispersity in combination with the highest melting point. Six carbon resonances were detected for all samples and were assigned according to *Randall*’s^[^ ^23^ ^]^ nomenclature for polyolefins. The carbon atoms closest to the stereo center (*CH*) are the most sensitive to the relative stereochemistry of neighboring monomer units and can be used to determine the stereoregularity. Unfortunately, a detailed pentad analysis was not possible due to the small difference between the individual signals. The mean difference between the carbon resonances for iso‐ and syndiotactic samples was about 0.1 ppm (Table S5 for all chemical shifts). Therefore, only qualitative assessments regarding the regularity could be made by gauging the overall shape of the resonances. The signals of the polymers synthesized with either **1b** or **2** are very similar with relatively sharp resonances and no clear additional signals visible, which suggests that these samples have few errors, although no approximate percentage can be given because the other signals might be obscured. Even though the polymers synthesized with **2** or **3** have quite low molecular weights compared to the ZN polymers, end group carbon signals could not be detected. The tacticity of the syndiotactic sample is considerably lower as small shoulders are visible in addition to the six main carbon resonances. Insertions of 89% *mmmm* have been reported for the propylene polymerization using **1b** at 40 °C.^[^ ^12^, ^13^ ^]^ **2** is known to give insertions of about 92% *mmmm* ^[^ ^24^ ^]^ after activation with MAO at 20 °C for liquid propylene polymerization, while **3** leads to about 86% *rrrr* insertions at 25 °C.^[^ ^15^ ^]^ The atactic sample (Table 1, Entry 10) was used to examine the pentad distribution with all possible pentads present (Figure 3b). The measurement was conducted with a 1 GHz NMR spectrometer (^13^C: 250 MHz) to ensure an adequate resolution. While a better resolution was certainly achieved, the pentads of the relevant carbon signals could not be fully distinguished. Only four overlapping signals could be observed for the αα‐carbon atom with an overall width of 0.9 ppm. All other carbon signals, including ^4^B_4_, show rather complex peak shapes. By comparison, the methyl and methylene carbon atoms of polypropylene give well resolved signals, which have an overall width of about 2.0 and 2.5 ppm, respectively (150 MHz, measured at 70 °C, C_2_D_2_Cl_4_).^[^ ^25^ ^]^

### Melting Behavior

**Figure 4:**
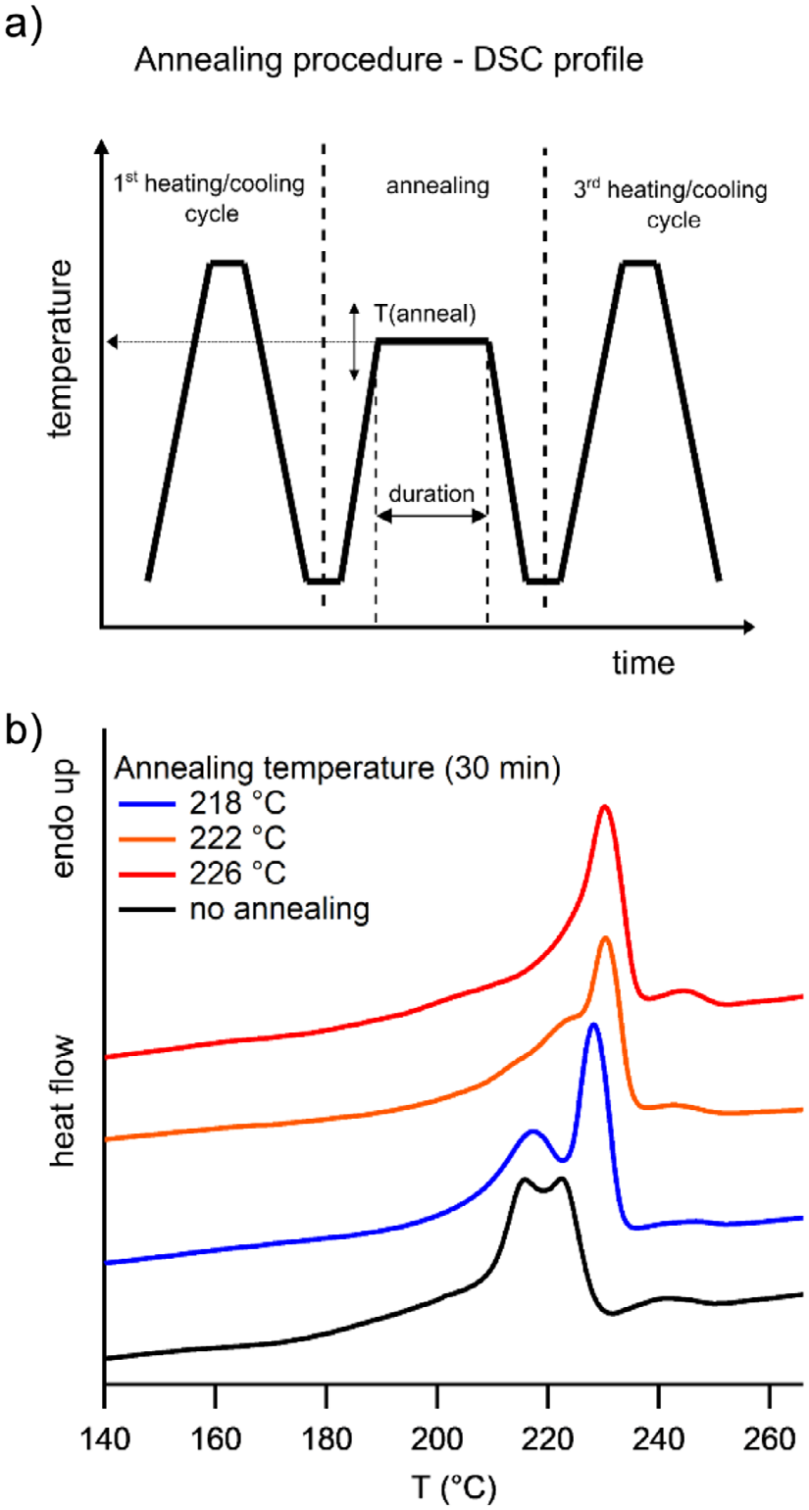
Differential scanning calorimetry (DSC) scans with and without annealing for *i*‐P4EH (Table 1, Entry 4). a) Schematic DSC temperature/time profile, heating/cooling rate: 10 K/min. b) Influence of the annealing temperature on the melting behavior (third heating is shown).

Important parameters which determine the melting characteristics include stereo‐ and regioregularity and chain length.^[^ ^18^ ^]^ The *i‐*P4EH samples reported in Table 1 exhibited melting endotherms between 206 and 226 °C. An exception is the polymer of Entry 1 (Table 1), which has no melting point. That melting behavior can be explained through a series of errors in the polymer chain. Lower reaction temperatures resulted in polymers with higher melting points. This behavior can be explained by higher *M*_w_ chains and fewer stereo/regio errors at low temperatures. Interestingly, *i*‐P4EH synthesized at 0 °C with **2** (*T*_m_: 222–224 °C) had a similar melting point to *i*‐P4MP (*T*_m_: 224 °C). This *T*_m_ for *i*‐P4MP is relatively low as it can melt up to 245 °C,^[^ ^26^, ^27^ ^]^ but is still in agreement with previous reports due to the low molecular weight and stereo/regio errors reported for the catalytic system.^[^ ^18^ ^]^ A *T*_m_ of 92 °C was observed for *i*‐P4EO. Regarding the syndiotactic samples (Table 1, Entries 8–9) prepared with **3** at 0 °C, only for Entry 8 a melting point (*T*_m_: 111 °C) was observed. This could be the consequence of a lower stereo‐ and regioregularity. Most polymers did not show a single melting endotherm (Figures 4, S8–S12, S24–S27 and S33). Similar behavior has been described for *i*‐P4MP and has been attributed to the following reasons; two or more crystal modifications, polymorphism (1), two or more crystal morphologies (2), two or more populations of lamellae with different thicknesses (3) or recrystallization phenomena during the heating (4).^[^ ^28^ ^]^ Different crystal morphologies can be ruled out because we performed multiple heating/cooling cycles to erase the thermal history. Regarding the structurally closely related *i*‐P4MP, only a single polymorph (polymorph I) crystallizes from the melt.^[^ ^26^, ^27^ ^]^ Several studies have shown that different populations of lamellae are present which melt at different temperatures.^[^ ^29^, ^30^, ^31^ ^]^ Furthermore, material melting at a lower melting point has sufficient time to recrystallize and remelt during the DSC scan. Annealing experiments were performed to further investigate the melting and crystallization behavior of *i*‐P4EH (see Figure 4). For this purpose, *i*‐P4EH (Table 1, Entry 2) was fully melted and cooled to erase its thermal history (first heating/cooling cycle) and annealed for 30 min at a temperature slightly below the highest melting endotherm. The molten material could then reorganize and recrystallize, thereby possibly thickening the remaining lamellae. After cooling, a third heating/cooling cycle was performed to completely melt the polymer. The annealing temperature *T*_anneal_ was increased stepwise from 218 to 226 °C (Figure 4). It was observed that the first melting endotherm (216 °C) decreased in intensity in favor of the second one (222 °C), which shifted to a slightly higher melting point of 228 °C with *T*_anneal_  =  218 °C. Increasing *T*_anneal_ resulted in a further increase of this melting point to 231 °C, while the first endotherm completely disappeared with *T*_anneal_  =  226 °C. A higher melting point was also reported for *i*‐P4MP annealing experiments and was explained as a consequence of lamellar thickening.^[^ ^29^, ^30^, ^31^ ^]^ Shorter or longer annealing durations (10 or 60 min) did not change the resulting heating endotherms (Figure S25). This indicates that the chain mobility is relatively high, and recrystallization is completed after 10 min. Since no glass transition temperature was observable by dynamic scanning calorimetry, we have used dynamic mechanical analysis (DMA). DMA possesses a 10 to 100 times higher sensitivity for glass transition temperature and made it possible to determine *T*_g_ to around 24 °C. (Figure S13) It must be noted that heating rate and frequency was adjusted to match the melting temperature from DSC to gain more evidence of the *T*_g_.^[^ ^32^ ^]^ X‐Ray diffraction of a quench cooled sample (Table S1, Entry 2) was measured. The degree of crystallinity was calculated by the method of Natta et al.,^[^ ^33^, ^34^ ^]^ which amounts to *X*_cryst._ = 17%. (Figure S14) Further characterization methods, such as IR spectroscopy (Figure S15), thermogravimetric analysis (Figure S28) were done for full characterization.

### Processing

**Figure 5:**
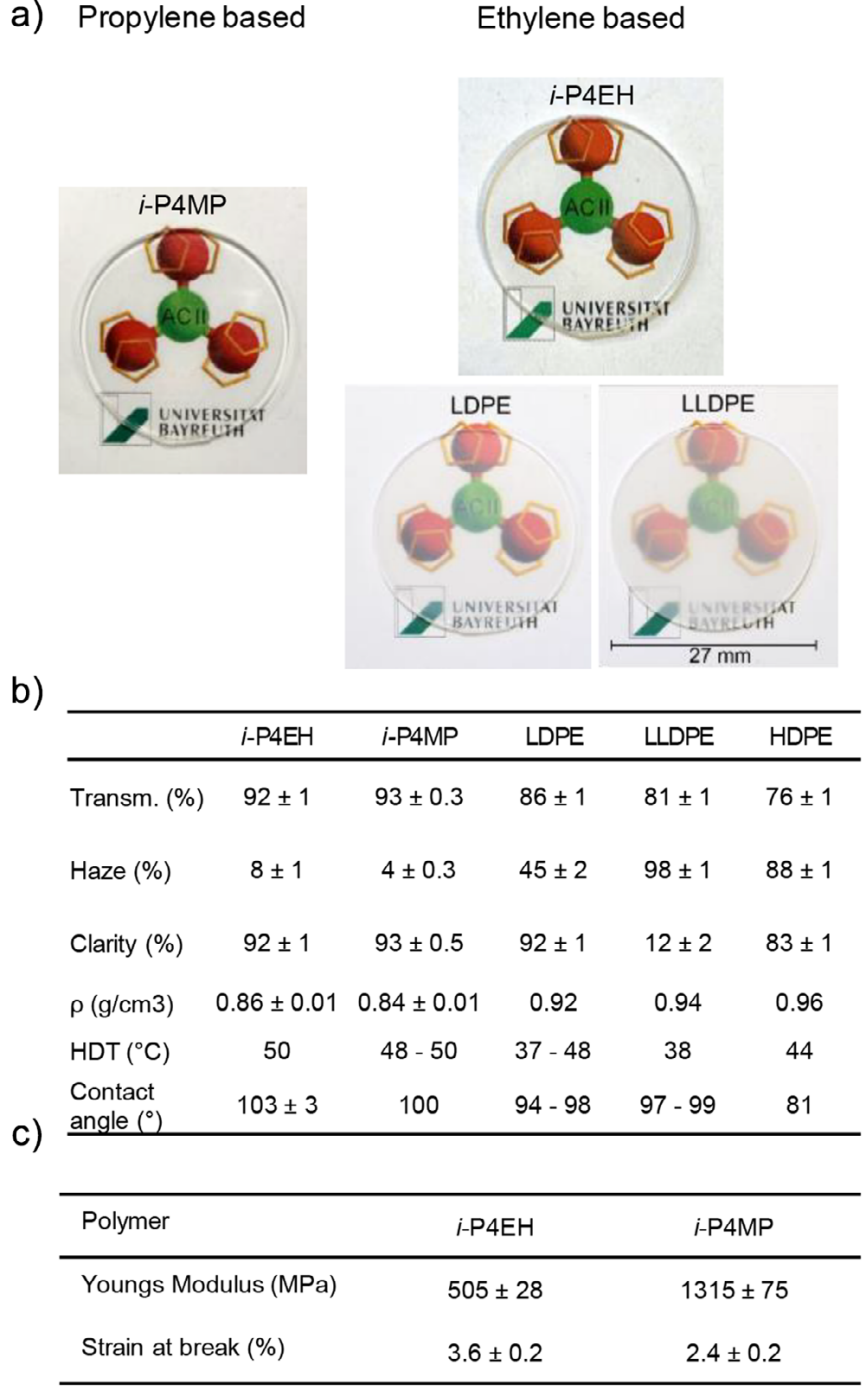
Comparison of *i*‐P4EH with *i*‐P4MP and various polyethylene materials. a) Visual demonstration of the polymer disks processed (*i*‐P4EH, *i*‐P4MP, LDPE, and LLDPE b) Optical characteristics of the polymer disks and density comparison of *i*‐P4EH and various polyethylene materials. For *i*‐P4MP literature values are given for better classification and comparison to *i*‐P4EH. Heat deflection temperature was compared to literature values.^[^ ^10^, ^35^, ^36^, ^37^, ^38^ ^]^ c) Mechanic properties obtained by stress strain characterization.

For thermal processing again only polymers produced with **1b** were chosen due to their good processability at high temperature. Polymers produced with the most productive catalyst **1c** however were difficult to melt. Injection molding proved unproblematic at 240 °C (Table 1, Entry 2) and disks (diameter: 27 mm, thickness: 1 mm) were prepared. A visual demonstration of its optical properties in comparison with various polyethylenes (LDPE, LLDPE, and HDPE (HDPE: Figure S40)) and *i*‐P4MP are shown in Figure 5. The *i*‐P4EH surpassed the polyethylenes investigated in terms of total transmittance, haze, and clarity. The density of the *i*‐P4EH was determined to be 0.86 ± 0.01 g cm^−3^, indicating that *i*‐P4EH is significantly lighter than other polyethylene materials and is equivalent to *i*‐P4MP which exhibits a density of 0.84, which is comparable to literature values. Stress strain characterization reveals that *i*‐P4EH is a brittle, hard material like *i*‐P4MP,^[^ ^10^, ^35^, ^36^, ^37^, ^38^ ^]^ having an Youngs modulus of 505 MPa and strain at break value of 14%. Heat deflection temperature (*T*_HDT_ = 50 °C at a load of 1.8 MPa) and VICAT softening temperature (*T*_VICAT_ = 37 °C at 50 N) for *i*‐P4EH were measured and comparable to literature values of *i*‐P4MP.^[^ ^35^, ^36^, ^37^, ^38^, ^39^ ^]^

## Conclusion

In summary, we report the synthesis of isotactic poly(4‐ethylhex‐1‐ene) (*i*‐P4EH), a highly transparent and processable ethylene‐based thermoplastic with an exceptionally high melting point above 220 °C and a very low density of 0.86 g cm^−^^3^. This material was prepared using Ziegler–Natta and metallocene catalysts under optimized conditions, yielding polymers with varying tacticities and molecular weights. Among the tested systems, Ziegler–Natta catalyst **1b** provided the best balance between productivity and polymer quality. *i*‐P4EH exhibits excellent optical properties (transmittance: 92%, haze: 8%, clarity: 92%). Annealing experiments revealed lamellar reorganization and melting point enhancement, highlighting the semi‐crystalline nature of *i*‐P4EH. These findings demonstrate the potential of *i*‐P4EH as a new class of high‐performance polyolefin with promising applications in areas requiring high thermal stability and optical clarity. Advantages of our material in comparison to *i*‐P4MP could be its ethylene based nature and its good solubility in organic solvents (even hexane) permitting solution processing.

## Conflict of Interests

The authors declare no conflict of interest.

## Supporting information



Supporting Information

## Data Availability

The data that support the findings of this study are available in the Supporting Informatation of this article.
