# Supplementary material for: A Highly Transparent Thermoplastic Synthesized from Ethylene that Melts Above 200 °C
Source: Angew Chem Int Ed Engl. 2025 Aug 17;64(40):e202505834. doi: 10.1002/anie.202505834 (PMC12462742; doi:10.1002/anie.202505834)
Supplement: Supplementary file 1 — Supporting Information [file ANIE-64-e202505834-s001.docx]

Supporting Information for

A highly transparent thermoplastic synthesized from ethylene that melts above 200 °C

Fabian Lukas, Andre Dickert, Winfried P. Kretschmer, Rhett Kempe

Corresponding author: Rhett Kempe, kempe@uni-bayreuth.de

Lehrstuhl Anorganische Chemie II – Catalyst Design, Sustainable Chemistry Centre,

Universität Bayreuth, 95440 Bayreuth, Germany

**The file includes:**

Materials and methods

Figs. S 1 to S 40

Tables S 1 to S 12

References

**General methods and materials**

All manipulations were performed with the rigorous exclusion of oxygen and moisture by using standard Schlenk type glassware on a dual-manifold Schlenk line and glovebox techniques (mBraun 120-G) with a high-capacity circulation (< 0.1 ppm O_2_) under an atmosphere of argon or nitrogen. Deuterated solvents were obtained from Eurisotop, degassed, distilled and stored over activated 3 Å molecular sieves prior to use. Solvents were dried and purified by distillation from LiAlH_4_, potassium, Na/K alloy or sodium benzophenone ketyl under argon atmosphere and stored over activated 3 Å molecular sieves before use. 1-Butene (2.5, Linde AG) was passed over scavenger columns (Supelco Big Moisture Trap Model 23991 and Supelco Big Supelpure^TM^ Model 503088) and ethylene (3.5, Linde AG) over columns of BASF R3-11 supported Cu oxygen scavenger and Al_2_O_3_ (Fluka). All other reagents and starting materials were purchased from commercial vendors with a purity of at least 97 % and used without further purification unless otherwise noted. Catylen C100 C-S300-068 (**1c**) was provided by Evonik. Commercial polymers for comparison purposes were received from SABIC (HDPE B5823) and LyondellBasell (LLDPE Lupolen 3621 MRM and LDPE Lupolen 1800P). In Table S1, technical properties of commercial polymers are provided.

**Table S 1: Common parameters of commercially available polymers.**

| **polymer** | **M_w_**  **(kg×mol^-1^)** | **Ð** | **T_m_**  **(°C)** | **MFR**  **(dg×min^-1^)** |
| --- | --- | --- | --- | --- |
| SABIC HDPE  B5823 (HDPE) | 678.2 | 54.0 | 133 | 0.16 (2.16 kg/ 230 °C)^a^ |
| LyondellBasell  Lupolen 3621MRM (LLDPE) | 84.4 | 5.1 | 114 | 7.5 (2.16 kg/ 190 °C)^b^ |
| LyondellBasell  Lupolen 1800P (LDPE) | 340.3 | 20.1 | 105 | 15 (2.16 kg/230 °C)^c^ |

Test method for MFR; ^a^HDPE: ASTM D 1238; ^b^LLDPE and LDPE ISO1133-1; LDPE

TiCl_4_ @ MgCl_2_^1^ **1a** including 9,9-Bis(methoxymethyl)fluorene^2^ **1b** as the internal donor (addition of 0.2 equivalents of donor during synthesis) were synthesized according to literature. Ti-content was determined via UV-Vis^3,4^ (Agilent Cary 60) to 5.1 wt‑%. Dichlorido[rac-ethylenebis(indenyl)]-zirconium(IV), Dichlorido[isopropylidene(3-cyclopentadiene-1-yl)fluoren-9-yl]zirconium(IV) and Dichloridocyclopentadienyl[tris(*tert*-butylphosphinylidene)amido]titanium(IV) Cp(*^t^*Bu_3_PN)TiCl_2_ were prepared according to literature.^5,6,7^ 4EH was synthesized according to our previous report^8^ and purified via multiple distillations (five section Snyder columns, purity: 99.2 mol-%, impurities: 0.3 mol‑% 1-octene and 0.5 mol-% 2-ethylhex-1-ene).

**Instruments**

*Nuclear magnetic resonance (NMR) spectroscopy:* ^1^H and ^13^C NMR spectra were collected on a Varian INOVA 300 (^1^H: 300 MHz, ^13^C: 75 MHz, 120 °C), a Bruker Avance III HD (^1^H: 500 MHz, ^13^C: 125 MHz, 23 °C) spectrometer equipped with a 5 mm CryoProbe^TM^ Prodigy BBO 500 S2 at or a Bruker Aeon 1GHz (^1^H: 1 GHz, ^13^C: 250 MHz, 23 °C). Chemical shifts (δ) reported in parts per million (ppm) and referenced internally to the residual solvent resonances. ^13^C assignment nomenclature is adopted from *Randall*.^9^ Multiplicities are given as follows: s: singlet, d: doublet, t: triplet, q: quartet, quint: quintet, m: multiplet, br: broad signal or combination thereof.

*Size-exclusion chromatography (SEC):* SEC analyses were performed on an Agilent (Polymer Laboratories Ltd.) PL-GPC 220 high temperature chromatographic unit equipped with DP and RI detectors, a guard and three linear mixed bed columns (Agilent Olexis). SEC analyses were carried out at 150 °C using 1,2,4-trichlorobenzene as the mobile phase. The samples were prepared by dissolving the polymer (0.1 wt-%) in the mobile phase solvent in an external oven and were run without filtration. The molecular weights of the samples were referenced to linear narrow HDPE standards (M_w_ = 110 – 430000 g mol^-1^, K = 40.6 and α = 0.725) and corrected with K = 19.4 and α = 0.81 for isotactic poly(4-methyl-1-pentene).

*Differential scanning calorimetry (DSC):* All DSC measurements were performed on a Mettler DSC 3+. Standard aluminum pans with a volume of 40 μL were used. The samples were measured in three cycles from 20 °C to 270 °C with a heating/cooling rate of 10 K/min and under a nitrogen atmosphere.

*Thermogravimetric analysis (TGA):* All TGA measurements were performed on a Mettler Toledo TGA / SDTA 851e. Standard aluminum pans with a volume of 40 μL were used. Samples were measured from 20 °C to 1000 °C with a heating rate of 10 K/min.

*Injection molding:* Injection molding was performed on a DSM Xplore Micro 12cc Injection Molding Machine. The polymer was filled into a preheated transfer line (up to 350 °C). After 5 min the polymer melt was injected into the mold (cooled to 30 °C) with 4 - 12 bar for 15 s depending on the polymer. The mold defines the geometry of the resulting discs with a diameter of 27 mm and 1 mm thickness.

*Optical measurements:* Transmittance, haze and clarity measurements were performed on a BYK Gardner haze-guard plus according to the norm ASTM D-1003 (transmittance: ratio of total transmitted light through the sample compared to the incident light; haze: ratio of scattered light through the sample with a deviation of at least 2.5 ° compared to the total transmitted light; clarity: ratio of scattered light through the sample with a deviation of not more than 2.5 ° compared to the sum of this narrowly scattered light and the transmitted parallel light).

*Density measurements:* Density measurements of polymer samples were performed on a Mettler Toledo weight balance (Archimedes principle; T = 22 °C, ρ_water_ = 0.9977 g/cm^3^).

Dynamic mechanical analysis (DMA): DMA measurements were performed on a DMTA III (TA instruments) at a heating rate of 2 K/min and a frequency of 2 Hz under nitrogen atmosphere.

*Compression Molding:* Dogbone samples for tensile test were manufactured at a Carver Type 2518 hotpress. Films with a thickness of 0.5 mm were compression molded at 250 °C for 4 minutes at a pressure of 5 tons. The polymer was mixed with 0.5 wt.-% of Irganox 1010 to prevent thermal degradation during processing.

*Tensile tests:* Tensile tests were performed on an Instron universal 5565 equipped with a video extensiometer. Dogbone shaped samples (0.5 mm (T) × 4 mm (W) x 15 mm (L)) were used. For calculation of Youngs Modulus a rate of 0.3 mm/min was applied until a strain of 0.5 % was achieved. After that the rate was increased to 1 mm/min. For each polymer, four samples were characterized

*X-ray diffraction:* Diffraction pattern was obtained using a Bragg-Benato type instrument (Empyrean Malvern Panalytical BV; The Netherlands) applying Cu Kα radiation (λ = 1.54187 Å). The diffractometer is equipped with an PIXcel-1D detector. Pawley Refinement was done by using GSAS-II ^10^

*VICAT and Heat deflection temperature* *(HDT)*: For VICAT and HDT measurements the heat distortion temperature tester CEAST was used. 2 Samples (10x4x10 mm for VICAT; 10x4x80 mm for HDT) were placed in a silicone oil bath and heated from 25 °C to 250 °C with a heating rate of 120 K/min. The samples were measured according to ISO309-B50 (VICAT) and ISO75-2A (HDT).

*Contact angle*: The surface water contact angle was determined using the Surftens Universal instrument (OEG, Germany). After setting the baseline, the droplet shape was fitted using the circular form of the Laplace–Young equation. The reported contact angle represents the average of five individual measurements.

**General polymerization procedures**

All polymerization reactions were carried out in 20 mL glass vials in an Ar-filled glovebox (mBraun 120G) on a magnetic stirring plate with integrated heater.

*Ziegler-Natta precatalysts* ***1a-c****:*

The vial was charged with toluene and 4EH (6 mmol) and was brought to the desired temperature. Subsequently, the precatalyst (15 μmol suspended in methylcyclohexane) and activator (0.2 mmol, stock solution in toluene) were added. V_total_ was 5 mL. After the reaction time, the mixture was poured into acidified EtOH (150 mL), the polymer filtrated, washed with excess EtOH and dried over night at 50 °C.

For preparation of polymer disks for optical characterisation, the polymer was dissolved in warm toluene (50 °C). An aqueous solution of Disodium-2,2′,2′′,2′′′-(ethane-1,2-diyldinitrilo)tetraacetate (Na_2_EDTA; 20 mmol/l; 200 ml)^11^ was added and the biphasic mixture was vigorously stirred overnight. The aqueous phase was then separated and washed with toluene. The organic phase was washed three times with water. After removal of the solvent under reduced pressure, the resulting polymer was dried over night at 50 °C. Prior to further processing, the polymer was ground to a fine powder.

*Homogeneous precatalysts* ***2****-****4****:*

The vial was charged with toluene and 4EH (6 mmol) and was brought to the desired temperature. Subsequently, scavenger, activator (stock solution in toluene) and the precatalyst (5 mm stock solution in toluene) were added. V_total_ was 5 mL. After the reaction time, the mixture was poured into acidified EtOH (150 mL), the polymer filtrated, washed with excess EtOH and dried over night at 50 °C.

**Table S 2: Polymerization experiments using 1a, 1b, 1c.^[a]^**

|  | | | | | | | | |
| --- | --- | --- | --- | --- | --- | --- | --- | --- |
| Entry | Cat. | Activator | T (°C) | $\frac{\text{n(Al)}}{\text{n(}\text{Ti}\text{)}}$ | Conversion (%) | M_w_ (kg/mol) | Ɖ | T_m_ (°C) |
| 1 | 1a | TIBA | 50 | 15 | 27 | 19.6 | 21.2 | n.d. |
| 2 | 1b | AlEt_3_ | 50 | 15 | 7 | 62.9 | 12.5 | 205 |
| 3 | 1b | AlClEt_2_ | 50 | 15 | 1 | 139.4 | 10.8 | 194 |
| 4 | 1b | TIBA | 50 | 15 | 22 | 248.8 | 11.0 | 226 |
| 5 | 1b | TIBA | 50 | 10 | 20 | 118.1 | 11.8 | n. d. |
| 6 | 1b | TIBA | 50 | 50 | 15 | 325.8 | 16.4 | n. d. |
| 7 | 1b | TIBA | 0 | 15 | 8 | n. d. | n. d. | 211 |
| 8 | 1b | TIBA | 30 | 15 | 25 | 395.7 | 17.8 | 221 |
| 9 | 1b | TIBA | 70 | 15 | 18 | 88.3 | 7.4 | 217 |
| 10^[b]^ | 1b | TIBA | 50 | 15 | 26 | 297.4 | 11.8 | n.d. |
| 11 | 1c | TIBA | 50 | 15 | 86 | 662.7 | 21.3 | 206 |

^[a]^ Reaction conditions: n(Ti) = 15 μmol, n(4EH) = 6 mmol, solvent: toluene, V(sum) = 5 mL, t = 30 min. ^[b]^ n(4MP) = 6 mmol instead of 4EH

**Figure S 1: HT-SEC of i-P4EH synthesized with 1a (Table S 2, Entry 1).**

**Figure S 2: HT-SEC of i-P4EH synthesized with 1b (Table S 2, Entries 2 and 3).**

**Figure S 3:** **HT-SEC of i-P4EH synthesized with 1b (Table S 2, Entries 4 and 5).**

**Figure S 4:** **HT-SEC of i-P4EH synthesized with 1b (Table S 2, Entries 6 and 8).**

**Figure S 5:** **HT-SEC of i-P4EH synthesized with 1b (Table S 2, Entry 9).**

**Figure S 6:** **HT-SEC of i-P4MP synthesized with 1b (Table S 2, Entry 10).**

**Figure S 7: HT-SEC of i-P4EH synthesized with 1c (Table S 2, Entry 11).**

**Figure S 8:** **Differential scanning calorimetry heating/cooling cycles of i-P4EH (left: comparison of different Ti/Al combinations, Table S 2, Entries 2-4; right: comparison of different reaction temperatures, Table S 2, Entries 4 and 7-9) from 30 °C to 270 °C. Heating/cooling rate: 10 K/min. Three cycles were performed, third heating is shown.**

**Figure S 9** **:** **Differential scanning calorimetry heating/cooling cycles of i-P4EH (Table S 2, Entry 1) from 30 °C to 270 °C. Heating/cooling rate: 10 K/min. Three cycles were performed, third cycle is shown.**

**Figure S 10:** **Differential scanning calorimetry heating/cooling cycles of i-P4EH (Table S 2, Entries 2 and 3) from 30 °C to 270 °C. Heating/cooling rate: 10 K/min. Three cycles were performed, third cycle is shown.**

**Figure S 11:** **Differential scanning calorimetry heating/cooling cycles of i-P4EH (Table S 2, Entries 4 and 7-9) from 30 °C to 270 °C. Heating/cooling rate: 10 K/min. Three cycles were performed, third cycle is shown.**

**Figure S 12: Differential scanning calorimetry heating/cooling cycles of i-P4EH (Table S 2, Entry 11) from 30 °C to 270 °C. Heating/cooling rate: 10 K/min. Three cycles were performed, third cycle is shown.**

***Figure S 13: Dynamic mechanic analysis of i-P4EH (Table S2, Entry 4) for determination of glass transition temperature*.**

**Figure S 14: Top: X-Ray diffraction pattern of i-P4EH (Table S2, Entry 4) Bottom: Area of background and distinct reflection area for determining the crystallinity.**

**Figure S 15: ATR IR spectrum of i-P4EH (Table S2, Entry 4); assignments were done according to Stivala and Gabbay and J. J. Samuel and Mohan.^12^**

**Figure S 16: Measurement for determining the Heat Deflection Temperature of i-P4EH (Table S2, Entry 4) according to DIN EN ISO75-2A (applied load 1.8 MPa) at a heating rate of 120 K/min**

**Figure S 17: VICAT measurement of i-P4EH (Table S2, Entry 4) for determining T_VICAT_. The measurement was done according to ISO309 B50 at a heating rate of 120 K/min.**


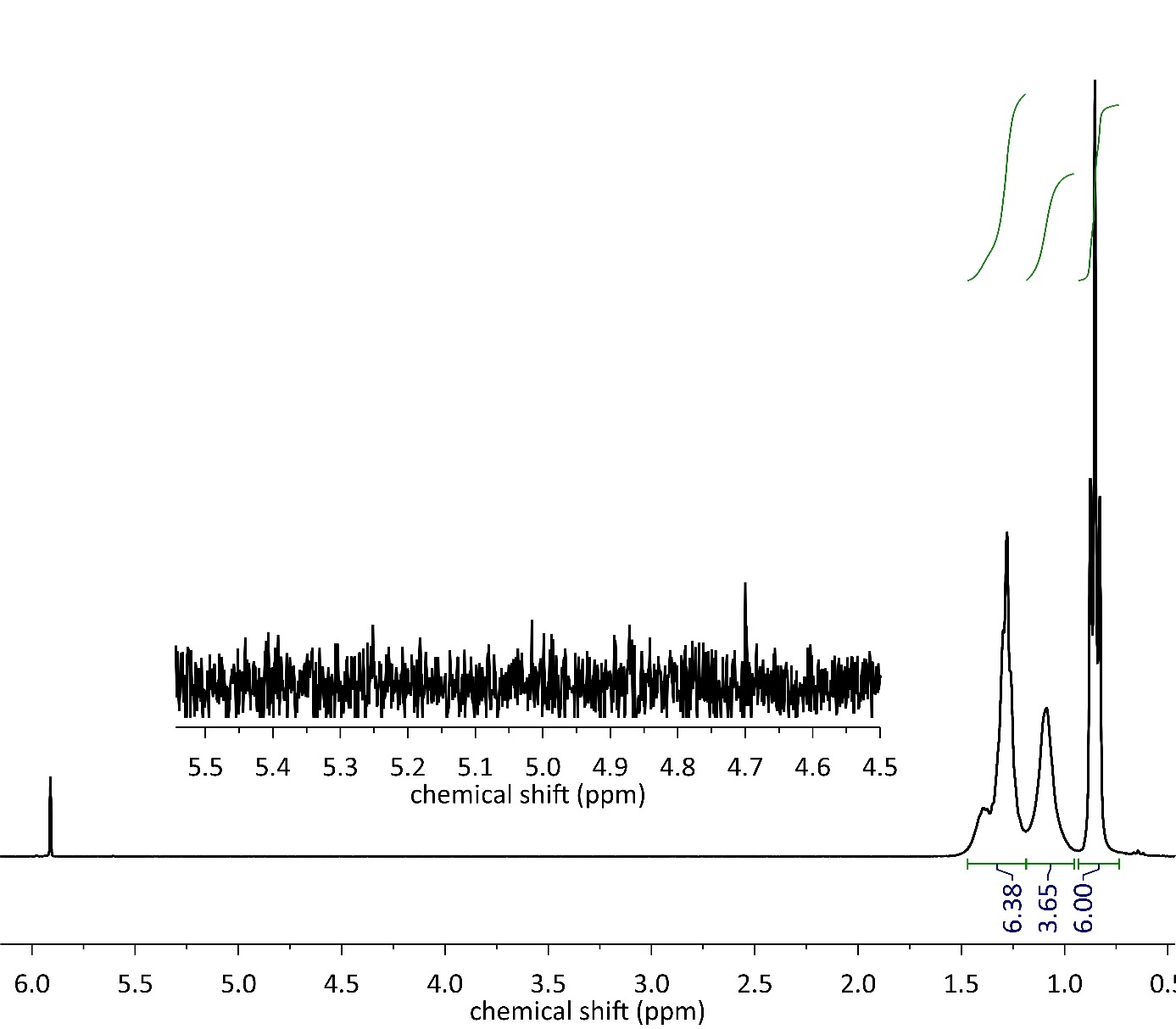


**C_2_D_2_Cl_4_**

**Figure S 18: ^1^H-NMR spectrum (300 MHz, 120 °C, C_2_D_2_Cl_4_) of i-P4EH which was produced using 1b (Table S 2, Entry 4). The inset shows the olefinic region and the absence of olefinic signals.**

isotactic poly(4-ethylhex-1-ene): ^1^H-NMR (300 MHz, C_2_D_2_Cl_4_, 393 K): δ = 0.86 (t, *J* = 7.4 Hz, 6H, H^1^), 0.94-1.18 (m, 4H, H^2^), 1.18-1.50 (m, 6H, H^3-6^) ppm. ^13^C-NMR (75 MHz, C_2_D_2_Cl_4_, 393 K): δ = 10.89, 26.39, 30.89, 38.42, 40.27, 42.56 ppm.

**Figure S 19:** **^13^C-NMR spectrum (75 MHz, 120 °C, C_2_D_2_Cl_4_ ) of i-P4EH which was produced using 1b (Table S 2, Entry 4).**

**Table S 3: Polymerization experiments using 2.^[a]^**

|  | | | | | | | | |
| --- | --- | --- | --- | --- | --- | --- | --- | --- |
| Entry | Monomer | Activator | T (°C) | $\frac{\text{n(Al)}}{\text{n(}\text{Zr}\text{)}}$ | Conversion (%) | M_w_ (kg/mol) | Ɖ | T_m_ (°C) |
| 12 | 4EH | TEAl / borate | 0 | 10 | 26 | 7.0 | 1.8 | 224 |
| 13 | 4EH | MAO | 0 | 500 | 30 | 4.5 | 1.6 | 223 |
| 14 | 4EH | TIBA / borate | 0 | 5 | 13 | 12.8 | 2.0 | n. d. |
| 15 | 4EH | TIBA / borate | 0 | 10 | 28 | 13.3 | 2.0 | 222 |
| 16 | 4EH | TIBA / borate | 0 | 50 | 27 | 11.5 | 2.0 | n. d. |
| 17 | 4EH | TIBA / borate | 30 | 10 | 45 | 12.4 | 2.0 | 216 |
| 18 | 4EH | TIBA / borate | 60 | 10 | 74 | 7.7 | 2.0 | 211 |
| 19 | 4MP | TIBA / borate | 0 | 10 | 54 | 13.8 | 1.9 | 224 |
| 20 | 4EO | TIBA / borate | 0 | 10 | 15 | 13.2 | 1.9 | 92 |

^[a]^ n(Zr) = 5 μmol, activator: 1.1 equiv. ammonium borate ([R_2_N(CH_3_)H]^+^[B(C_6_F_5_)_4_]^-^, R = C_16_H_33_ to C_18_H_37_) or methylalumoxane (MAO), scavenger and alkylating agent: TEAl, TIBA or MAO, t = 7 h, n(monomer) = 6 mmol, solvent: toluene, V(sum) = 5 mL.

**Figure S 20:** **HT-SEC of i-P4EH synthesized with 2 (Table S 3, Entries 12 and 13).**

**Figure S 21:** **HT-SEC of i-P4EH synthesized with 2 (Table S 3, Entries 14 and 15).**

**Figure S 22:** **HT-SEC of i-P4EH synthesized with 2 (Table S 3, Entries 16 and 18).**

**Figure S 23:** **HT-SEC of polymers synthesized with 2 (Table S 3, Entries 19 and 20).**

**Figure S 24:** **Differential scanning calorimetry heating/cooling cycles of i-P4EH synthesized with 2 (top left: comparison of different Zr/activator combinations, Table S 3, Entries 12, 13 and 15 top right: comparison of different reaction temperatures, Table S 3, entries 15, 17 and 18; bottom left: comparison of different monomers, Table S 3, entries 15, 19 and 20).**

**Figure S 25: DSC** **annealing experiments of using the polymer of Entry 15 (Table S3). The sample was annealed at the specified temperature for a specified time after a regular heating/cooling cycle had been performed. The heating afterward is shown from 30 °C to 270 °C. Heating/cooling rate: 10 K/min. Left: Variation of annealing temperature. Right: Variation of annealing time.**

**Figure S 26:** **Differential scanning calorimetry heating/cooling cycles of i-P4EH synthesized with 2 (Table S 3, Entries 12, 13, 15 and 17) from 30 °C to 270 °C. Heating/cooling rate: 10 K/min. Three cycles were performed, third cycle is shown.**

**Figure S 27:** **Differential scanning calorimetry heating/cooling cycles of polymers synthesized with 2 (Table S 3, Entries 18-20) from 30 °C to 270 °C. Heating/cooling rate: 10 K/min. Three cycles were performed; third cycle is shown.**

**Figure S 28: Thermogravimetric analysis curve of i-P4EH (Table S 3, Entry 15). N_2_ atmosphere (left) and air (right) with heating rate: 10 K/min.**


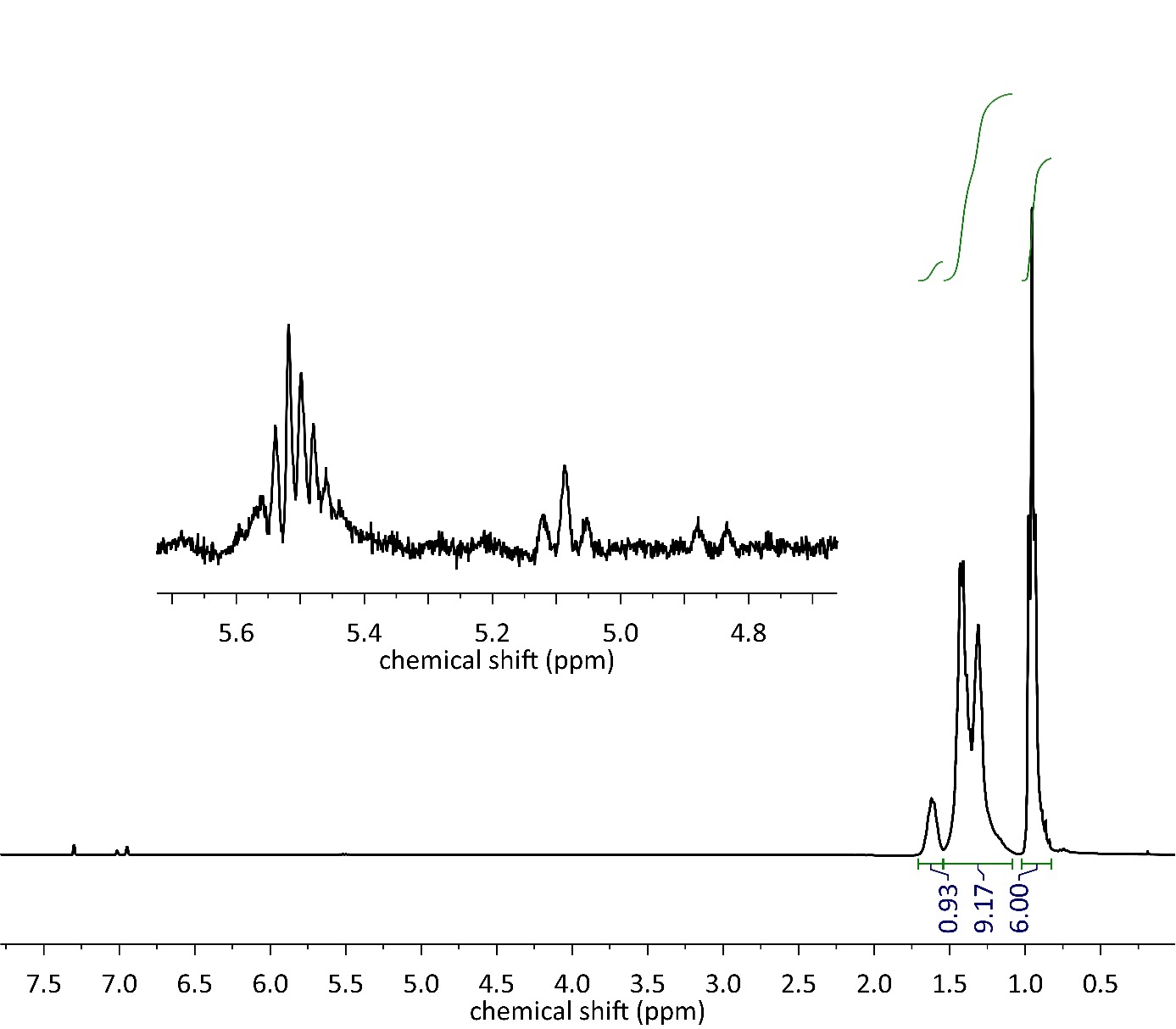


**C_6_D_5_Br**

**Figure S 29: ^1^H-NMR spectrum (300 MHz, 120 °C, C_6_D_5_Br) of i-P4EH which was produced using 2 (Table S 3, Entry 15). The inset shows the olefinic region. Vinylidene and internal olefin signals could be detected.**

isotactic poly(4-ethylhex-1-ene): ^1^H-NMR (300 MHz, C_6_D_5_Br, 393 K): δ = 0.95 (t, *J* = 6.6 Hz, 6H, H^1^), 1.04-1.53 (m, 9H, H^2^), 1.53-1.70 (m, 1H, H^5^) ppm. ^13^C-NMR (75 MHz, C_2_D_2_Cl_4_, 393 K): δ = 10.89, 26.40, 30.90, 38.42, 40.27, 42.57 ppm.


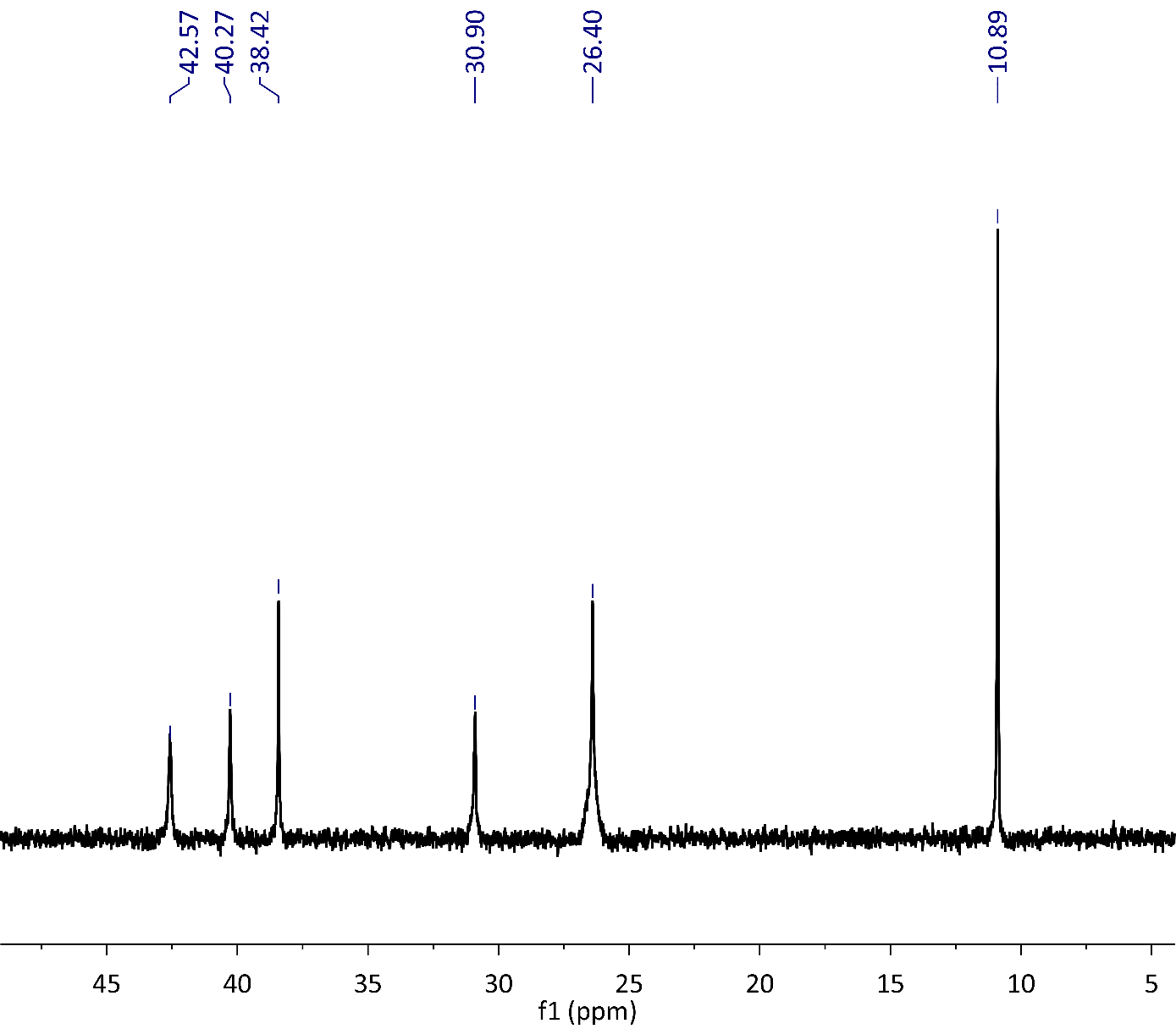


αα

^1^B_4_

^2^B_4_

CH

^3^B_4_

^4^B_4_

**Figure S 30: ^13^C-NMR spectrum (75 MHz, 120 °C, C_2_D_2_Cl_4_) of i-P4EH which was produced using 2 (Table S 3, Entry 15).**

**Table S 4: Polymerization experiments using 3.^[a]^**

|  | | | | | | |
| --- | --- | --- | --- | --- | --- | --- |
| Entry | Precatalyst | T (°C) | Conversion (%) | M_w_ (kg/mol) | Ɖ | T_m_ (°C) |
| 21 | **3** | 0 | 10 | 10.7 | 1.8 | 111 |
| 22 | **3** | 30 | 33 | 8.4 | 1.9 | / |
| 23 | **3** | 60 | 8 | 4.8 | 1.6 | / |

^[a]^ Reaction conditions: activator: 1.1 equiv. ammonium borate, alkylating agent and/or scavenger: 10 equiv. TIBA, t = 7 h, n(monomer) = 6 mmol, solvent: toluene, V(sum) = 5 mL.

Precatalyst **3**: n(Zr) = 5 μmol.

**Figure S 31:** **HT-SEC of s-P4EH synthesized with 3 (Table S 4, Entries 21 and 22).**

**Figure S 32:** **HT-SEC of s-P4EH synthesized with 3 (Table S 4, Entry 23).**

**Figure S 33:** **Differential scanning calorimetry heating/cooling cycles of s-P4EH synthesized with 3 (Table S 4, Entry 21) from 30 °C to 270 °C. Heating/cooling rate: 10 K/min. Three cycles were performed, third cycle is shown.**


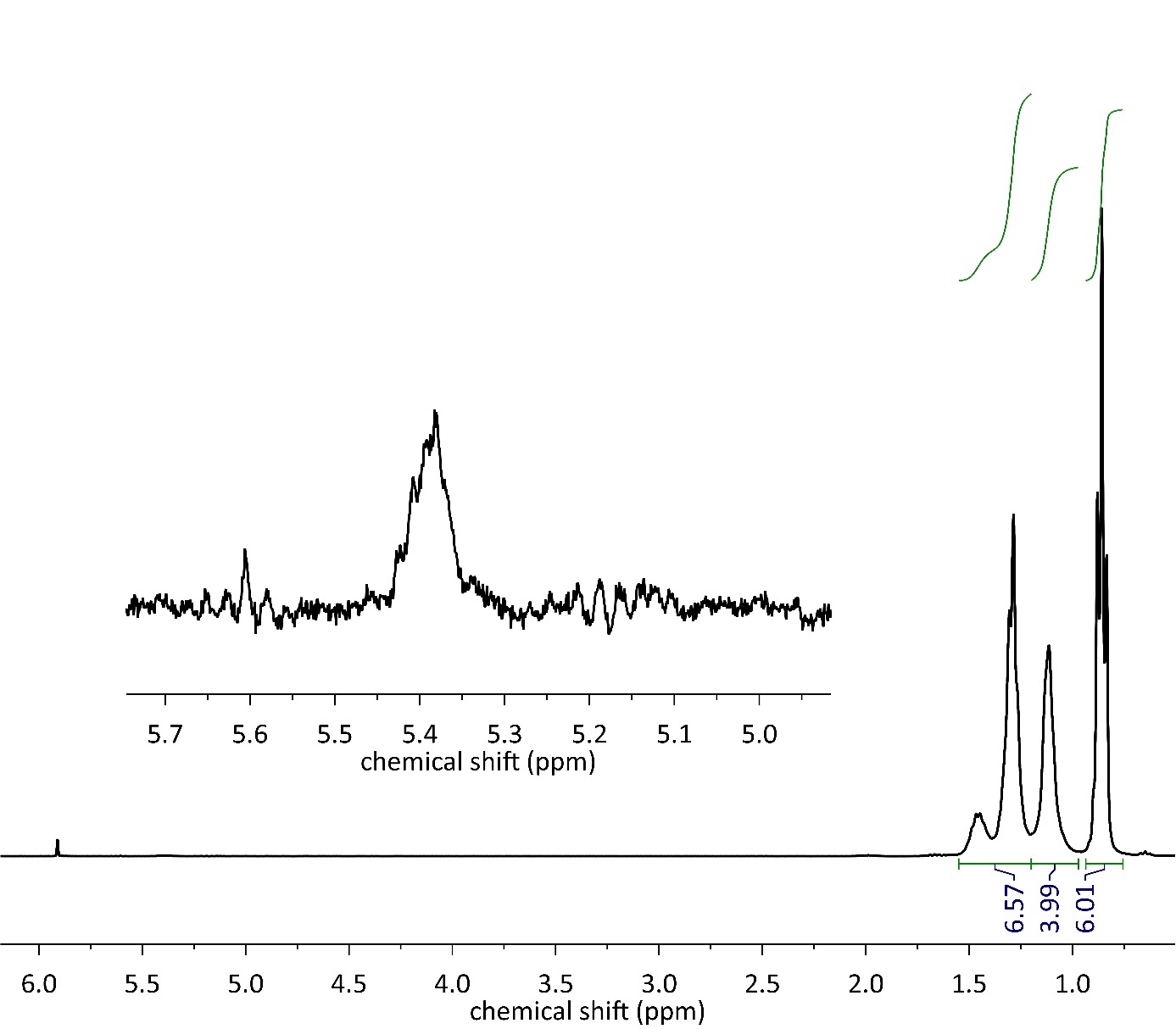


**C_2_D_2_Cl_4_**

**Figure S 34:** **^1^H-NMR spectrum (300 MHz, 120 °C, C_2_D_2_Cl_4_) of s-P4EH which was produced using 3 (Table S 4, Entry 21). The inset shows the olefinic region. Internal olefin signals were detected.**

syndiotactic poly(4-ethylhex-1-ene): ^1^H-NMR (300 MHz, C_2_D_2_Cl_4_, 393 K): δ = 0.86 (t, *J* = 6.7 Hz, 6H, H^1^), 0.98-1.19 (m, 4H, H^2^), 1.19-1.55 (m, 6H, H^3-6^) ppm. ^13^C-NMR (75 MHz, C_2_D_2_Cl_4_, 393 K): δ = 10.92, 26.30, 31.03, 38.36, 40.14, 42.76 ppm.


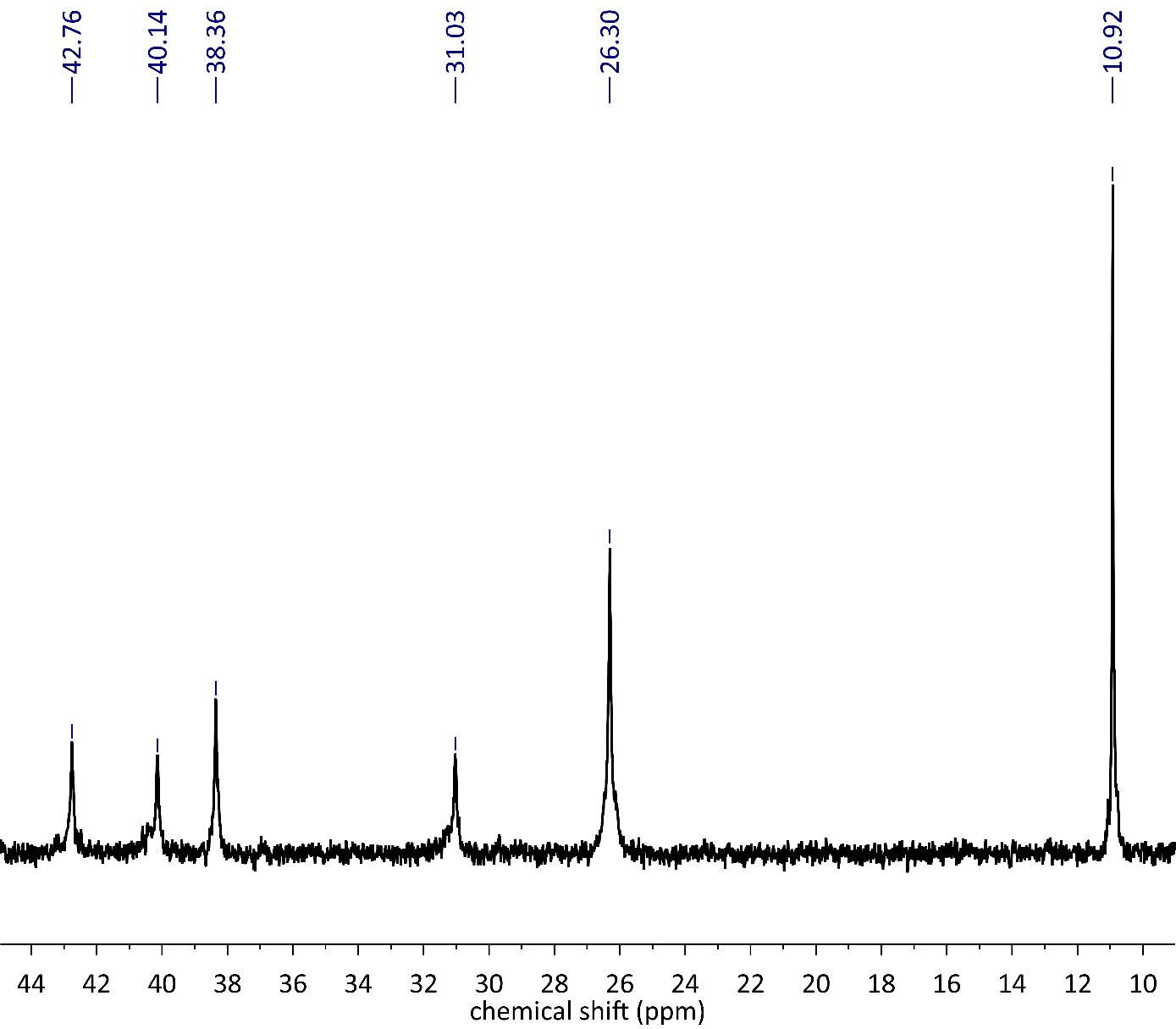


αα

^3^B_4_

CH

^2^B_4_

^1^B_4_

^4^B_4_

**Figure S 35:** **^13^C-NMR spectrum (75 MHz, 120 °C, C_2_D_2_Cl_4_) of s-P4EH which was produced using 3 (Table S 4, Entry 21).**

**Table S 5: ^13^C NMR chemical shifts (120 °C, C_2_D_2_Cl_4_) of P4EH produced with catalyst systems based on 1b, 2 and 3.**

| Carbon atom | Chemical shift (ppm) | | | Δ(iso/syndio) (ppm) |
| --- | --- | --- | --- | --- |
|  | Isotactic / **1b** | Isotactic / **2** | Syndiotactic / **3** |  |
| ^1^B_4_ | 10.89 | 10.89 | 10.92 | 0.03 |
| ^2^B_4_ | 26.39 | 26.40 | 26.32 | 0.08 |
| ^3^B_4_ | 38.42 | 38.42 | 38.36 | 0.06 |
| ^4^B_4_ | 42.56 | 42.57 | 42.76 | 0.20 |
| αα | 40.27 | 40.27 | 40.14 | 0.13 |
| CH | 30.89 | 30.90 | 31.03 | 0.14 |

***Table S 6: Preparation of an atactic sample using 4 for NMR comparison purposes.***

|  | | | | | | | |
| --- | --- | --- | --- | --- | --- | --- | --- |
| Entry | Activator | T (°C) | t (h) | Conversion (%) | M_w_^SEC^ (kg/mol) | Ɖ | T_m_ (°C) |
| 24 | ammonium borate | 60 | 7 | 18 | 1.6 | 1.9 | / |

^[a]^ Reaction conditions: n(Ti) = 20 μmol, activator: 1.1 equiv.; n(4EH) = 6 mmol, solvent: toluene, V(sum) = 5 mL, scavenger/alkylating agent: TIBA; n(TIBA) = 200 μmol.

The reaction mixture was extracted with 5 mL of diluted HCl_aq_ and dried under reduced pressure at 80 °C for 24 h.


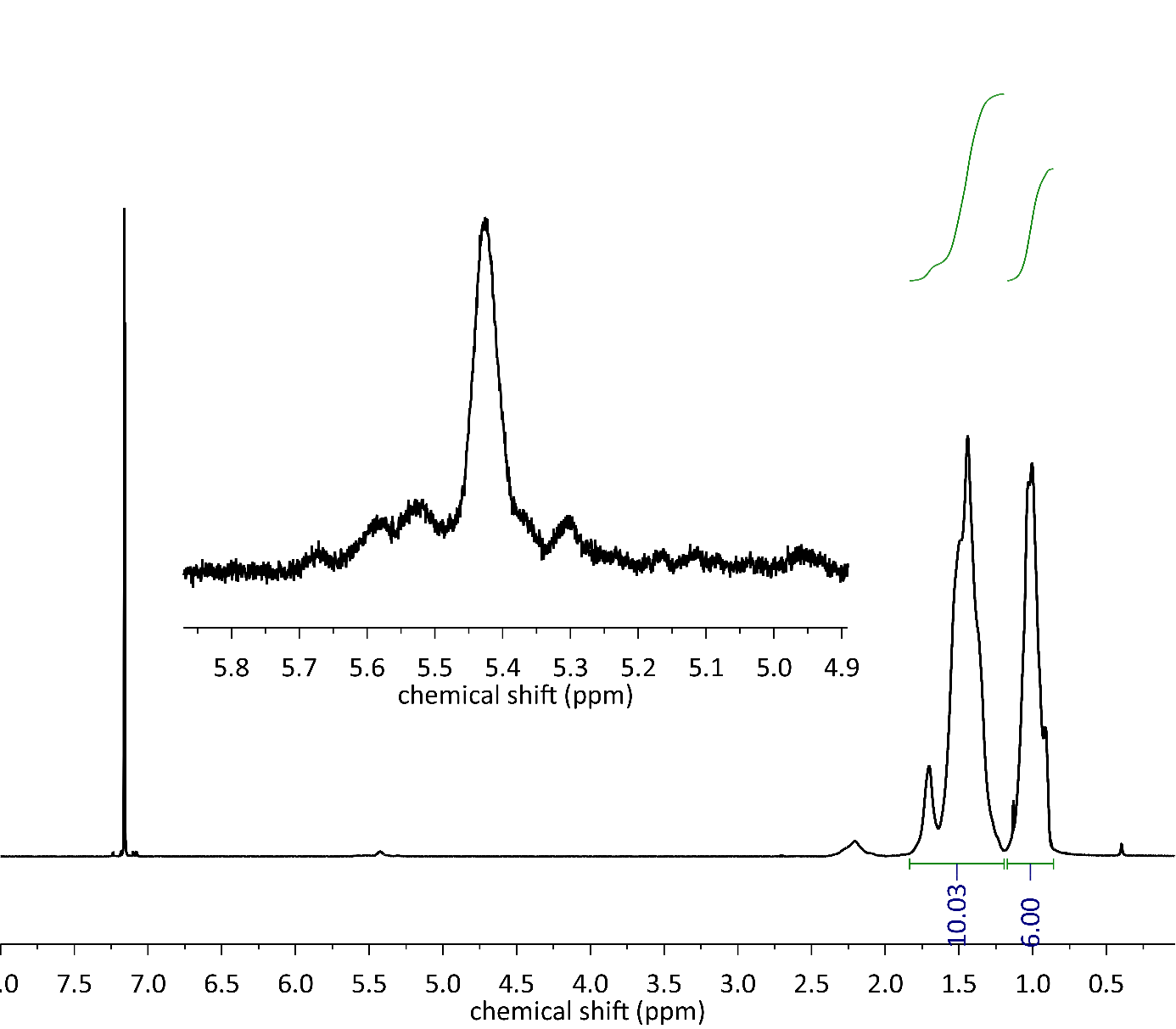


**C_6_D_6_**

**Figure S 36:** **^1^H-NMR spectrum (1 GHz, 23 °C, C_6_D_6_) of a-P4EH which was produced using 4 (Table S 6, Entry 24). The inset shows the olefinic region. Internal olefin signals were detected.**


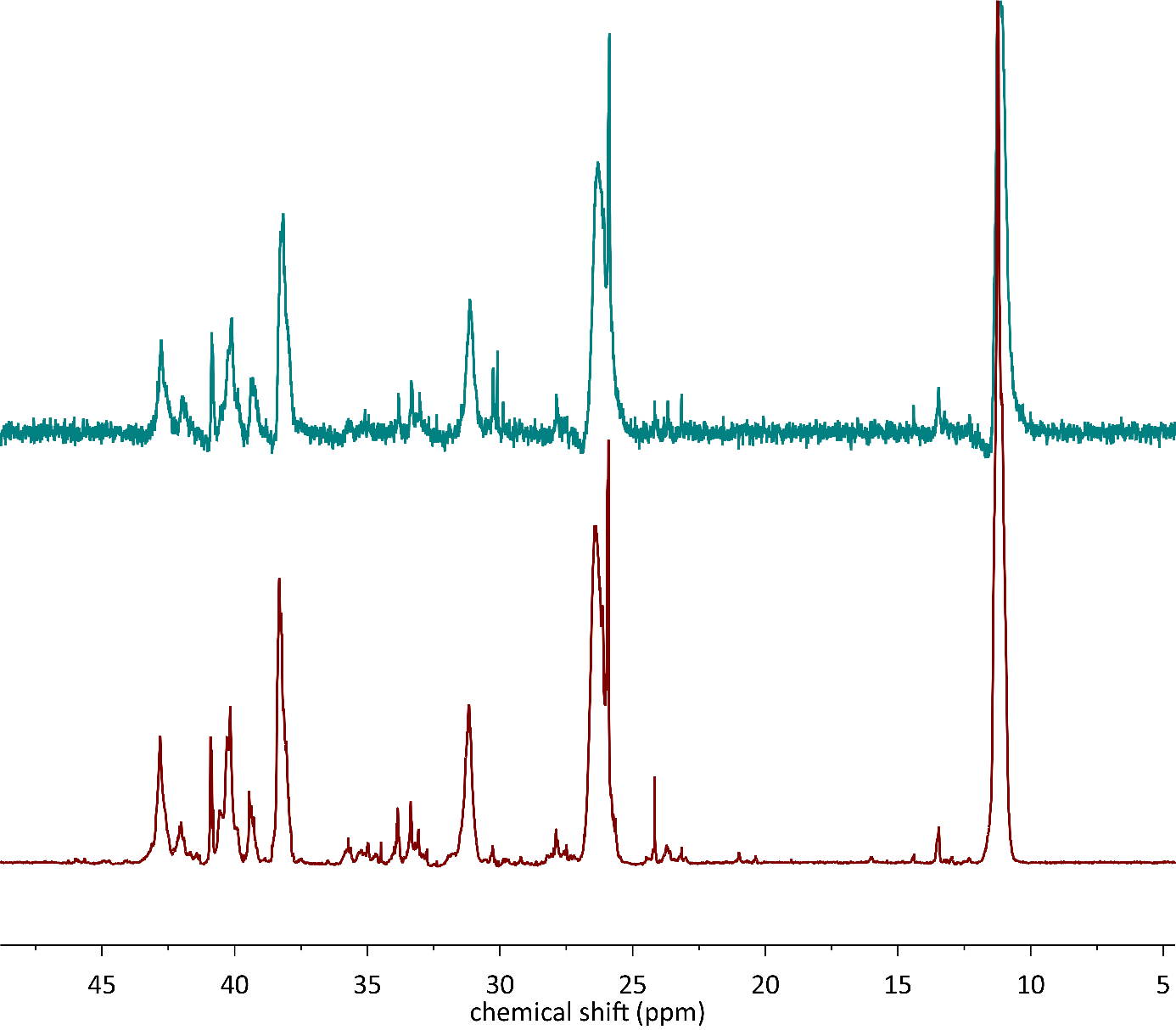


**125 MHz**

**250 MHz**

**Figure S 37:** **^13^C-NMR spectra comparison of a-P4EH which was produced using 4 (Table S 6, Entry 24) with different magnetic field strengths. Top: 125 MHz, 23 °C, C_6_D_6_. Bottom: 250 MHz, 23 °C, C_6_D_6_.**

**Table S 7:** **^13^C NMR chemical shifts (23 °C, C_6_D_6_) of atactic P4EH produced with catalyst system based on 4.**

| Carbon atom | Chemical shift (ppm) |
| --- | --- |
| ^1^B_4_ | 10.73 – 11.58 |
| ^2^B_4_ | 25.61 – 26.86 |
| ^3^B_4_ | 37.81 – 38.58 |
| ^4^B_4_ | 42.42 – 43.06 |
| αα | 39.80 – 40.71 |
| CH | 30.78 – 31.72 |

**Table S 8: Transmittance, haze and clarity of various polyethylene-based materials and i-P4EH. Polymer disks: diameter: 27 mm, thickness: 1 mm.**

| Polymer / sample number | | Transmittance (%) | Haze (%) | Clarity (%) |
| --- | --- | --- | --- | --- |
| *i*-P4EH | 1 | 92.1 | 7.9 | 92.8 |
|  | 2 | 92.8 | 8.2 | 92.7 |
|  | 3 | 91.5 | 7.7 | 92.4 |
|  | 4 | 92.5 | 8.5 | 92.1 |
|  | 5 | 92.9 | 9.4 | 91.0 |
|  | Mean | 91.5 ± 0.4 | 8.3 ± 0.6 | 92.2 ± 0.6 |
| *i*-P4MP | 1 | 93.5 | 4.0 | 95.2 |
|  | 2 | 93.9 | 4.4 | 94.3 |
|  | 3 | 93.2 | 3.6 | 94.5 |
|  | 4 | 92.9 | 3.9 | 95.8 |
|  | 5 | 93.2 | 4.2 | 94.6 |
|  | mean | 93.3 ± 0.3 | 4.0 ± 0.3 | 94.8 ± 0.5 |
| LDPE  Basell Lupolen 1800P | 1 | 86.1 | 47.7 | 92.0 |
|  | 2 | 84.9 | 42.9 | 90.1 |
|  | 3 | 86.3 | 42.7 | 93.1 |
|  | 4 | 86.5 | 42.2 | 92.2 |
|  | 5 | 86.3 | 45.0 | 92.3 |
|  | Mean | 86.0 ± 0.7 | 44.1 ± 2.3 | 91.9 ± 1.2 |
| LLDPE  Basell Lupolen 3621 | 1 | 80.7 | 97.8 | 14.5 |
|  | 2 | 80.8 | 97.7 | 11.1 |
|  | 3 | 80.8 | 97.4 | 11.9 |
|  | 4 | 81.7 | 97.7 | 8.9 |
|  | Mean | 81.0 ± 0.5 | 97.7 ± 0.2 | 11.6 ± 2.4 |
| HDPE  Sabic B5823 | 1 | 75.4 | 87.0 | 84.3 |
|  | 2 | 76.1 | 87.5 | 81.8 |
|  | 3 | 75.7 | 88.2 | 83.1 |
|  | 4 | 76.0 | 87.9 | 81.9 |
|  | 5 | 76.0 | 88.0 | 82.0 |
|  | Mean | 75.8 ± 0.3 | 87.7 ± 0.5 | 82.6 ± 1.1 |

**Table S 9: Density measurements of i-P4EH (Table S 2, Entry 4).**

| *i*-P4EH sample number | σ (g/cm^3^) |
| --- | --- |
| 1 | 0.864 |
| 2 | 0.862 |
| 3 | 0.863 |
| 4 | 0.864 |
| Mean | 0.86 ± 0.01 |

**Table S 10: Density measurements of i-P4MP (Table S 2, Entry 10)**

| *i*-P4MP sample number | σ (g/cm^3^) |
| --- | --- |
| 1 | 0.844 |
| 2 | 0.848 |
| 3 | 0.851 |
| 4 | 0.831 |
| Mean | 0.84 ± 0.01 |

**Table S 11: Contact angle measurements of i-P4EH (Table S 2, Entry 4)**

| *i*-P4EH sample number | (°) |
| --- | --- |
| 1 | 99.58 |
| 2 | 103.80 |
| 3 | 107.32 |
| 4 | 104.85 |
| 5 | 102.92 |
| 6 | 103.19 |
| Mean | 103.6 ± 2.3 |

**Figure S 38: Stress strain diagram of i-P4EH (Table S2, Entry 4).**

**Figure S 39: Stress strain diagram of i-P4MP (Table S2, Entry 10).**

**Table S 12: Summary of parameters obtained by characterization of i-P4EH and i-P4MP by stress strain experiments**

| Entry | E-Modulus  [MPa] | Strain at break  [%] | Stress at break  [mm] |
| --- | --- | --- | --- |
| *i*-P4EH | 505 ± 28 | 3.6 ± 0.2 | 8.0 ± 0.8 |
| *i*-P4MP | 1315 ± 75 | 2.4 ± 0.2 | 16 ± 0.7 |

**Figure S 40: Visual demonstration of the HDPE disks processed**

**References**

^1^ B. G. Song, S.-K. Ihm, *J. Appl. Polym. Sci.* **2014**, *131***,** 40536-40543.

^2^ D. Xu, Z. Liu, J. Zhao, S. Han, Y. Hu, *Macromol. Rapid Commun.* **2000**, *21*, 1046–1049.

^3^ G. Schwarzenbach, J. Muehlebach, K. Mueller, *Inorg. Chem*. **1970**, *9*, 2381–2390.

^4^ U. C. Makwana, K. J. Singala, R. B. Patankar, S. C. Singh, V. K. Gupta, *J. Appl. Polym. Sci.* **2012**, *125*, 896–901.

^5^ F. R. W. P. Wild, M. Wasiucionek, G. Huttner, H. H. Brintzinger, *J. Organomet. Chem.* **1985**, *288*, 63.

^6^ J. A. Ewen, R. L. Jones, A. Razavi, J. D. Ferrara, *J. Am. Chem. Soc*. **1988**, *110*, 6255.

^7^ D. W. Stephan, J. C. Stewart, F. Guérin, R. v. H. Spence, W. Xu, D. G. Harrison, *Organometallics* **1999**, *18*, 1116.

^8^ F. Lukas, P. A. Simon, T. Dietel, W. P. Kretschmer, R. Kempe, *Adv. Sci.* **2024**, 2405653.

^9^ J. C. Randall, *J. Macromol. Sci., Polym.* **1989**, *29*, 201–317.

^10^ B.H. Toby, R.B. Von Dreele, *J. Appl. Cryst***.** **2013**, *46*, 544–549

^11^ (a) J. Barnes, S. Lippy, P. Zheng, R. Rajaram, WO2012009639A2, **2012**. (b) N. L. Cull, C. L. Aldridge, US3098845A, **1963**. (c) H. J. Kablitz, H. Strametz, US4476297A, **1984**.

^12^ (a) S. M. Gabbay, S. S. Stivala, *Polymer* **1976**, *17*, 121. (b) E. James Jebaseelan Samuel, S. Mohan, *Spectrochim Acta A* **2004**, *60*, 19.
